# Extensive patient-to-patient single nucleus transcriptome heterogeneity in pheochromocytomas and paragangliomas

**DOI:** 10.3389/fonc.2022.965168

**Published:** 2022-08-15

**Authors:** Peter Brazda, Cristian Ruiz-Moreno, Wout L. Megchelenbrink, Henri J. L. M. Timmers, Hendrik G. Stunnenberg

**Affiliations:** ^1^ Princess Máxima Center for Pediatric Oncology, Utrecht, Netherlands; ^2^ Department of Molecular Biology, Faculty of Science, Radboud University, Nijmegen, Netherlands; ^3^ Department of Precision Medicine, University of Campania Luigi Vanvitelli, Naples, Italy; ^4^ Department of Internal Medicine, Radboud University Medical Center, Nijmegen, Netherlands

**Keywords:** pheochromocytoma, neuroendcrine tumor, single cell RNA seq, transcriptome, heterogeneity, SDHB, RET, paraganglinoma

## Abstract

Pheochromocytomas (PC) and paragangliomas (PG) are rare neuroendocrine tumors with varied genetic makeup and are associated with high cardiovascular morbidity and a variable risk of malignancy. The source of the transcriptional heterogeneity of the disease and the underlying biological processes that determine the outcome of PCPG remain largely unclear. We focused on PCPG tumors with germline SDHB and RET mutations, which represent distinct prognostic groups with worse or better prognoses, respectively. We applied single-nuclei RNA sequencing (snRNA-seq) to tissue samples from 11 patients and found high patient-to-patient transcriptome heterogeneity in neuroendocrine tumor cells. The tumor microenvironment also showed heterogeneous profiles, mainly contributed by macrophages of the immune cell clusters and Schwann cells of the stroma. By performing non-negative matrix factorization, we identified common transcriptional programs active in RET and SDHB, as well as distinct modules, including neuronal development, hormone synthesis and secretion, and DNA replication. Similarities between the transcriptomes of the tumor cells and those of the chromaffin- and precursor cell types suggests different developmental stages at which PC and PG tumors appear to be arrested.

## Introduction

Pheochromocytomas (PC) and sympathetic paragangliomas (PG) are rare neuroendocrine tumors that originate from chromaffin cell-related populations located inside or outside the adrenal glands, respectively. PCPG is associated with significant morbidity and mortality (1). The current therapy of choice is surgical resection; however, the disease can be associated with a lifelong risk of tumor persistence or recurrence (2).

A plethora of genes have been reported to be responsible for a diverse hereditary background in up to 40% of PCPG (3, 4). PCPG is divided into two major classes based on bulk transcriptional and genomic profiles. Tumors in class 1 are predominantly extra-adrenal and display germline mutations in the succinate dehydrogenase complex (SDHB, SDHC, and SDHD, collectively referred to as SDHx), the most common form of PCPG. SDHx tumors have the worst prognosis, with a 30–70% risk of metastasis or recurrence (5). Class 2 PCPG, detected in 5% of hereditary PCPGs, is comprised of germline and/or somatic mutations of the RET proto-oncogene and has a better prognosis.

In this study, we exploited recent advances in single-nuclei RNA-seq to compare the gene expression landscapes of PCPG with SDHB and RET germline mutations, explore transcriptional heterogeneity, and gain insight into the molecular basis of their different prognoses.

## Materials and methods

### Preparation of single-nuclei suspensions

Previously selected tissue blocks were transferred to the RadboudUMC biobank and stored at -80°C. Nuclei were prepared from the frozen tissues under RNAse-free conditions. Briefly, samples were cut into ~7 mm pieces and kept on dry ice. The pieces were minced in a pre-cooled douncer in 500uL ice-cold Nuclei EZ lysis buffer 5x with pestle-A and 10x with pestle-B. The suspension was passed through a 70 µm cell strainer, washed with 1.5 mL cold Nuclei EZ Lysis, and incubated on ice for 5 min. The lysate was washed in Nuclei wash/resuspension buffer (1xPBS completed with 1% BSA and 0.2U/ul RNAsin Plus (Promega, #N2611) and passed through a 40 µm cell strainer. The nuclei were stained with DAPI. To exclude doublets and debris from the final mix and to precisely determine the number of loaded nuclei, we used FACS. A total of 15000 nuclei were sorted into a pre-cooled tube containing the RT-mix (RT-reagent + TSO + Reducing agent B) Immediately before loading the mix to one lane of the Chromium chip, 8.3 ul RT-enzyme was added to the mix, according to the standard protocol of the Chromium Single Cell 3’ kit (v2). All steps for library preparation were performed according to the manufacturer’s protocol. Paired-end sequencing was performed to sequence the prepared libraries using an Illumina NextSeq sequencer.

### Single-cell RNA-seq data processing and quality control

Raw sequencing data were converted into FASTQ files using bcl2fastq. Reads were aligned to the human genome reference sequence (GRCH38) and counted using STAR. The CellRanger (10X Genomics) analysis pipeline was used for sample demultiplexing and single-cell gene counting to generate the gene-cell expression matrix for each library. The gene expression matrix was then processed and analyzed using the *Seurat* package in R. To filter out low-quality cells, we first removed cells (nuclei) with less than 10% or more than 250% of the mean gene count (*nFeature_RNA*) within each individual library. The cell count and gene count information for the single-cell datasets of the PCPG samples are listed in **Table 1**.

**Table 1:** Clinical information and snRNAseq quality parameters of processed/analyzed samples.

'PPGL_' ID	Age	Date of operation	Mutation group	Mutation	Location	Metastatic	number of captured nuclei (after filtering)	gene count (after filtering)
66	51	2012	RET	C611Y	adrenal gland	no	7063	1697
100	48	2012	RET	C634R	adrenal gland	no	3602	3110
180	30	2013	RET	C611Y	adrenal gland	no	6829	2696
269	47	2012	RET	C818S	adrenal gland	no	6437	1658
370	71	2017	RET	C611Y	adrenal gland	no	3175	2113
373	32	2017	SDHB	exon3del	adrenal gland	yes	3836	767
77	28	1998	SDHB	exon3del	bladder	yes	3772	1916
92	21	1988	SDHB	N109H	retroperitoneal	no	6232	1916
313	31	2009	SDHB	IVS4+1G>A	retroperitoneal	no	5448	1171
266	15	2012	SDHB	R115*	mediastinal	no	1526	1419
227	25	2012	SDHB	C192R	mediastinal	no	2948	2111

See also in **Table S1**.

### Dimensionality reduction, clustering and visualization

Data were normalized, scaled to 10000 counts and log-transformed using the NormalizeData function of the Seurat package. Principal component analysis was performed on the scaled data with the 4000 most variable genes. Using the 15 first principal components, we calculated a UMAP representation of the data for visualization and calculated clusters using the *FindNeighbors* and *FindClusters* functions with the resolution parameter set to 0.3. Marker genes that differentiated between clusters were identified using the FindAllMarkers function.

To identify the cell types, we used sets of well-established markers and annotated each cell type based on their average expression. Differentially expressed (marker) genes were determined using the *FindAllMarkers* function and were required to be expressed in at least 25% of the cells in a cluster with a minimal log expression difference of 0.25 between clusters. The gene sets were further filtered for p-values (< 0.05) and log2FoldChange >|1.2|.

### Inferred CNV analysis from snRNA-seq

Large-scale copy number variations (CNVs) were inferred from single-nuclei gene expression profiles using the *inferCNV* package (6) using the i3 HMM parameter, a window size of 101 genes and the “cluster_by_groups” parameter is true. To identify distinct chromosomal gene expression patterns in neuroendocrine cells, all other cells were set as the “reference” cells. CNVs in the reference cells would still detectable.

### Expression programs of intra-tumoral heterogeneity

We applied non-negative matrix factorization (NMF) using the *RunNMF* function of the swne (7) package to extract transcriptional programs of malignant cells from each sample. We set the number of factors as 28 for each sample. For each of the resulting factors, we considered the top 50 genes with the highest NMF scores as the characteristics of the given factor. We used the *AddModuleScore* function in the Seurat package to evaluate the degree to which individual cells express a certain pre-defined expression program and thus determine the scores. All tumor cells were scored according to the 280 NMF programs. Hierarchical clustering of the scores for each program using Pearson correlation coefficients as the distance metric and Ward’s linkage revealed ten correlated sets of metaprograms. The gene list of the 10 meta-programs is shown in **Table S5**.

### Logistic regression for similarity calculation

To measure the similarity of a target single-cell transcriptome to a reference single-cell dataset, we used the logistic regression method described previously (8). Briefly, we trained a logistic regression model with elastic net regularization (α = 0.6) on the reference training set. We then use this trained model to infer a similarity score for each cell in the query dataset for each cell type in the reference data. The predicted logits were averaged within each cluster or sample group of the query dataset.

## Results

**Figure 1:**
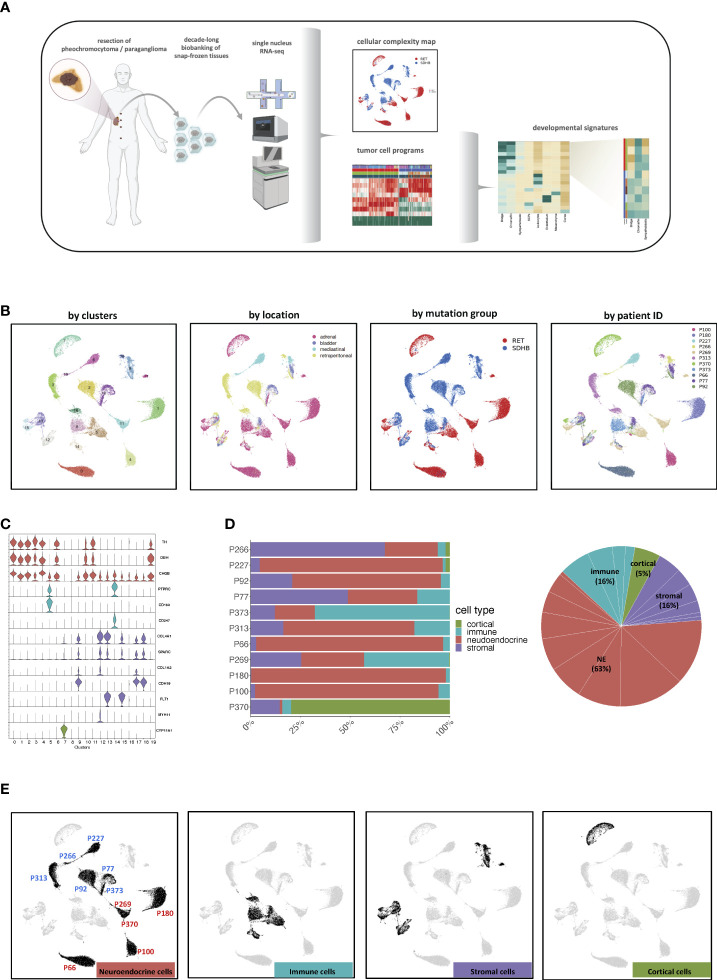
**(A)** Graphical abstract of the study. Created with BioRender.com. **(B)** UMAP visualization of all 50 868 cells grouped according to their cluster annotation and colored by their clusters, location of origin, mutation group, or patient ID. **(C)** Violin plots displaying the expression levels of canonical markers of representative cell types. **(D)** Distribution of cell types across the merged dataset and per sample. **(E)** UMAP visualization of all 50,868 cells, highlighting the cells annotated as the main cell types. The UMAP clusters of NEs were also marked by their most representative patient IDs.

We performed single-nucleus transcriptomic profiling (snRNA-seq) on resected tumor tissues from 11 treatment-naïve patients to generate a comprehensive PCPG atlas (**Figure 1A**). Molecular diagnoses revealed germline RET and SDHB mutations in 5 and 6 patients, respectively (**Table S1**). All RET-PCPG samples were retrieved from the adrenal gland, whereas the SDHB-PCPG tumors were collected from various locations, including the bladder, adrenal gland, retroperitoneal, and mediastinal areas.

### Cell type composition of PCPG tissue

Stringent quality filtering yielded 50 868 nuclei, with an average of 1 800 genes per nucleus (Methods, **Table S1**; **Figure S1A**). The merged expression profiles were compressed into a 2D-coordinate system using uniform manifold approximation and projection for dimension reduction (UMAP). The cells were grouped into 20 clusters and annotated based on their location, mutation group, and patient ID (**Figure 1B**; **Table S2**).

Based on canonical marker genes, we identified three major groups of cell types: neuroendocrine (NEs (markers TH, DBH, and CHGB)), immune (PTPRC, CD163, and CD247), and stromal (COL4A1 and COL1A2) cells (**Figure 1C**). The analysis of cluster 7 revealed that it originated almost exclusively from one donor (P370) and was characterized by elevated expression of typical adrenocortical rather than adrenomedullary marker genes, such as CYP11A1 and CYP11B1 (**Figures 1C, D**; **Figures S1C, D**). Hence, cells from donor P370 were considered non-representative and excluded from downstream analysis. Neuroendocrine cells (NEs) represented the largest cell fraction (63%, clusters 0, 1, 2, 3, 4, 6, 10, 11, 16, 19), followed by stromal (16%, Clusters 9, 12, 13, 15, 17, 18) and immune cells (16%, Clusters 5, 8, 14, 16) (**Figure 1D**). Most NE clusters consisted of cells from a single patient (**Figures S1B–D**). However, cells in the tumor microenvironment (TME) occupied shared UMAP territories (**Figure 1E**). Based on these observations, we decided not to apply batch correction in subsequent analyses to maintain the biological heterogeneity.

To obtain a more detailed insight into the cellular complexity of the TME, immune and stromal cells were subselected separately for further analyses. Annotation of immune cells (**Figure S2A**) resulted in the assignment of macrophages, which are the major components of the immune TME (9) (expressing CD163, CDSF1R, TGFBI), followed by T-cells (CD247, IL7R, TCF7), and B-cells (MS4A1, BLK, BANK1) (**Figure S2B**). Macrophages are the most heterogeneous immune cells, which could be related to tissue-specific transcriptional programs, as they are widely known to exert context-specific functions (10, 11) (**Figure S2A**, *arrows*). However, adrenal macrophages (colored green) derived from the same location but from different tumor samples were very different. (**Figure S2A**, *blue and red arrows*). This suggests that the macrophage transcriptome not only has a strong locational component but also a tumor type-specific component. The T and B cells originating from different locations and mutation groups appeared rather similar as they clustered together. Finally, the annotation of the stromal group (**Figures S2C, D**) revealed Schwann cells (expressing SOX6, CDH19, and NRXN1), endothelial cells (FLT1, PECAM1, and PTPRB), and fibroblasts (TAGLN, ACTA2, and COL1A1).

The numbers of individual immune and stromal cell populations were deemed too small for an in-depth analysis and were not further investigated.

### RET and SDHB tumor cells display chromosomal aberrations

**Figure 2:**
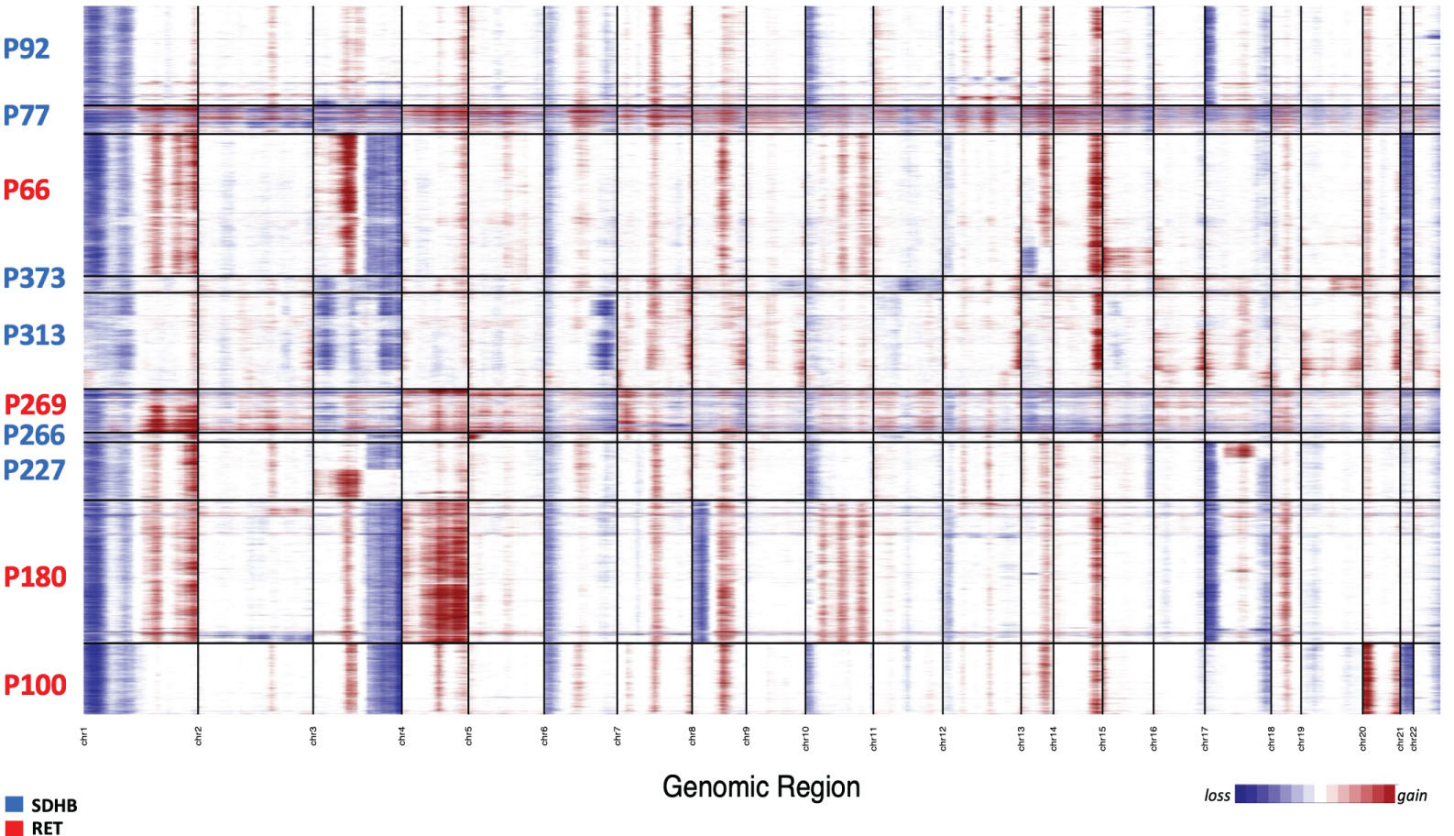
Heatmap of inferred CNVs of NE cells (immune clusters and stromal clusters were applied as reference). The patient IDs are colored by the mutation groups.

We explored inferred Copy Number Variation (iCNV) to determine large-scale somatic chromosomal changes (**Figure 2**). Immune- and stromal cells served as ‘reference’ in the assumption that large CNVs do not occur in the non-malignant. In agreement with published whole-genome sequencing profiles of PCPG tissue (12–15), segmental loss in the p-arm of chromosome 1 (1p) was present in all examined tumors regardless of the mutation type. Loss of 1p was not found in the TME cells confirming the assignment of the neuroendocrine cells as PCPG tumor cells. In addition, we observed widespread loss in other chromosomes for example the 3q and 6p arms as well as patient-specific aberrations such as loss in chr21 and gain in 1q, 3q, 13q and 14q (**Figure 2**). Apart from a few exceptions (RET-PCPG P66) we found different iCNV patterns in chr13 and chr15 in a subset of the tumor cells; in P227 (SDHB-PCPG) we identified small variations in chr3 and chr17 but observed few intra-individual heterogeneities.

In contrast to the extensive inter-individual and tumor-specific genomic aberrations, the inferred genomic profiles of tumor cells within each tumor population were largely homogeneous, suggesting that the genome remained largely stable following an initial catastrophic event.

### Transcription programs separate RET- and SDHB PCPG tumor cells

**Figure 3:**
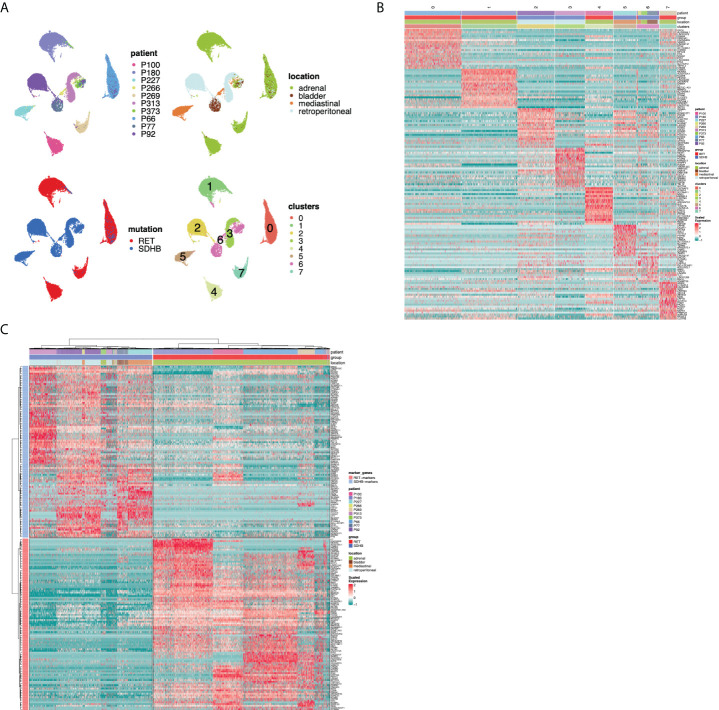
**(A)** UMAP visualization of the PCPG tumor cells subcluster after re-clustering (no batch-correction), annotated by patient ID, tumor location, mutation group and cluster. **(B)** Hierarchical clustering of differentially expressed genes for UMAP clusters across PCPG tumor cell subclusters. **(C)** Hierarchical clustering of differentially expressed genes for RET and SDHB mutation groups (sn-markers) across PCPG tumor cells.

To assess the inter-tumoral heterogeneity between RET and SDHB PCPG tumor cells, we selected and re-clustered tumor cells. With this finer-grained resolution, we identified UMAP clusters that consisted of cells mostly from one patient. This impinged on both the UMAP plots annotated by patient IDs (**Figure 3A**) and the heatmap annotation of the hierarchical clustering of the top20 cluster markers (**Figure 3B**; **Table S3**), reinforcing the strong inter-individual heterogeneity observed in the iCNV analysis. Selecting the tumor cells allowed us to determine the genes that were differentially expressed between the mutation groups (**Figure 3C**; **Table S4**). The newly identified markers were associated with either overlapping KEGG pathways (‘nervous system development’) or with gene ontology terms related to the secretory function of chromaffin cells (‘ion channel activities’, data not shown) (16).

**Figure 4:**
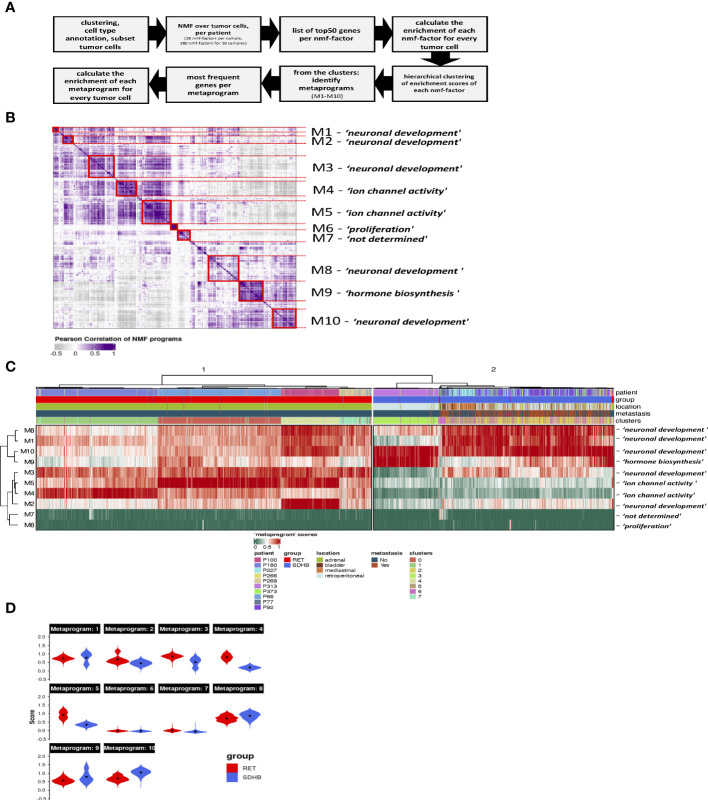
**(A)** Steps of NMF-analysis in PCPG. **(B)** Heatmap showing the correlation and hierarchical clustering of the 280 factors calculated in our NMF-analysis of the tumor cells individual samples, across all mutation groups. Metaprograms are numbered M1-M10 and annotated by their representative ontology terms. **(C)** Heatmap showing scores of PCPG tumor cells for the 10 metaprograms identified from NMF analysis of individual samples (clusters from **Figure 3A**). **(D)** Violin plots showing scores of PCPG tumor cells for the 10 metaprograms identified from the NMF analysis grouped per mutation group (black dots mark the mean, *Wilcoxon p<2.2e-16* within each Metaprogram).

We aimed to determine the transcriptional programs that are active across tumor cells and then identify the programs that are differentially enriched between RET and SDHB tumors. We applied non-negative matrix factorization (NMF) (7) to the sub-selected tumor cells to determine the full transcriptional spectrum behind the intratumoral heterogeneity and to extract the most representative biological processes in the tumor cells. First, we identified 28 active transcriptional programs in PCPG tumor cells from each sample based on their transcriptional profiles at the single-cell level (**Figure 4A**). The signature enrichment of these 280 programs was calculated for each individual tumor cell of the entire dataset. Next, based on the enrichment scores, we hierarchically clustered the programs and identified ten metaprograms (**Figure 4B**). Genes were ranked according to their frequency of presence within one metaprogram. These metaprograms spanned a narrow range of functions (**Table S5**), including neuronal development (metaprograms and their most representative genes: M1: BMPR1B, ROBO1; M2: NRG1, NTNG1; M3: FGF14, ROBO1; M8: SYT1, CTNNA2; M10: HDAC9, RORA), ion channel activity (M4: RYR2, PDE4B; M5: CACNA2D3, CHRM3), hormone synthesis (M9: TH, GCH1), and proliferation (M6: BRIP1, HELLS). Metaprogram seven (M7) was not associated with a significant ontology term.

Hierarchical clustering of metaprogram-scored cells revealed two major clusters separating RET from SDHB tumor cells (**Figure 4C**). The subclusters within the RET branch were segregated among the patient samples. In the SDHB branch, however, only sample P313 formed a discrete subcluster, whereas the tumor cells of other SDHB patients formed mixed subclusters. Surprisingly, SDHB tumor cells (originating from various anatomical locations) were less heterogeneous than their RET counterparts (originating from the adrenal gland).

The most pronounced differences in the average enrichment scores between RET (cluster 1) and SDHB (cluster 2) clusters were evident in the M2, M3, M4, and M5 metaprograms (**Figure 4D**). The ‘ion channel activity’ of the M4-M5 metaprograms is highly enriched among the RET tumor cells indicating a high secretory activity of the adrenal RET-pheochromocytoma tumor cells. The M9 ‘hormone synthesis’ program was more enriched among the SDHB tumor cells, mainly due to patient P313. A very small fraction of cells scored high for the ‘proliferation’ (M6) metaprogram, revealing the low but appreciable proliferative capacity of PCPG tumor cells. Several metaprograms were associated with the ontology terms of ‘neuronal development’ and were shared in both branches of the tumor group separation.

In summary, NMF analysis revealed two main transcriptional programs in PCPG that separate RET from SDHB tumor cells. Genes associated with ion channel activity (secretion) were enriched in RET tumors. We also observed that ‘neuronal development’ was a highly represented transcriptional program in both PCPG tumor cells.

### PCPG tumor cells display early adrenal developmental signatures

**Figure 5:**
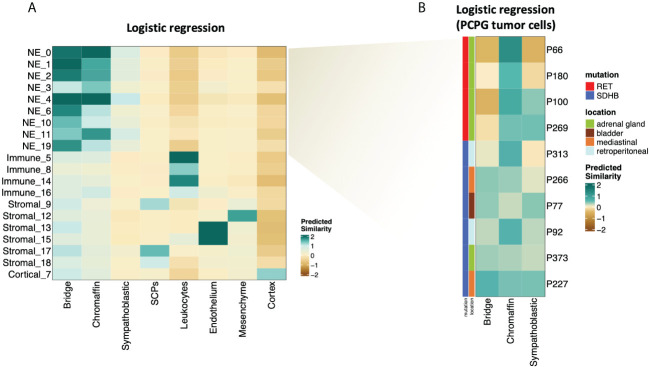
**(A)** Heatmap showing similarity scores (logistic regression and logit scale) of the signatures of developing cell types from (17) (fetal adrenal dataset) (x axis) to PCPG cells (y axis). **(B)** Heatmap showing similarity scores (logistic regression and logit scale) of the signatures of developing adrenal cell types from (17) (fetal adrenal dataset) (x-axis) to PCPG tumor cells by patient (y-axis).

The NMF analysis revealed several metaprograms that were associated with neuronal development but showed different enrichment scores among the mutation groups. This implies that the developmental signature is an important element of the tumor cell transcriptome; however, the differences between the mutation groups were not reflected in the ontology terms. To shed light on the developmental aspects of SDHB and RET-PCPG tumors, we compared the transcriptome of the cell types identified in the developing human adrenal gland (8-21 weeks (17) with PCPG tumors. We applied logistic regression and calculated the probability scores for cell type matches (**Figure 5A**). The analysis revealed that tumor cells were most similar to the cells at the junction of sympathoblast and chromaffin cells, called the ‘bridge cells’ (18). The cell types in the PCPG microenvironment showed high similarity with their normal cell counterparts in the developmental adrenal gland dataset.

The difference between SDHB and RET-PCPG became even more evident when the tumor cells of each patient were compared to chromaffin developmental cell types (**Figure 5B**). Logistic regression confirmed that the RET-PCPGs were more similar to the reference chromaffin cells, while the SDHB-PCPGs scored highest with both the chromaffin and bridge cell types, suggesting an earlier developmental state.

## Discussion

We performed snRNA-seq and mapped the transcriptional landscape of PCPG to investigate tumor heterogeneity and identify the transcriptomic programs associated with the mutation group of the tumor. We explored transcriptional heterogeneity by analyzing the transcriptomic profiles of 50 868 single nuclei from 11 patients (counting all cell types from five RET- and six SDHB PCPG tissue samples). This is the first study to reveal PCPG heterogeneity and the consequences of germline mutations at the single-cell level.

Neuroendocrine cells, the largest population in the dataset, were identified as tumor cells based on marker genes, and in particular, by inferring copy number variations from gene expression levels (19). The iCNV profiles revealed two important features. First, the lack of tumor cell sub-clusters within patients suggests a single initial catastrophic event that led to the birth of the tumor cells. Second, apart from very few recurring aberrations, we identified patient-specific iCNV patterns, marking the level of inter- and intratumoral heterogeneity in the PCPG cellulome, which provided a challenge for tumor classification.

To identify the patterns of the single-nuclei transcriptomic profiles based on tumor cells, we applied NMF, an unsupervised learning approach that is employed to approximate high-dimensional datasets in a reduced number of meaningful components (7, 20, 21). The analysis of single-nuclei transcriptomes of >30,000 tumor cells resulted in 10 metaprograms across the entire tumor set. The transcription programs related to ion channel activity (transmembrane transport) separated the SDHB and RET tumor cells. Based on biochemical analysis of plasma, urinary, and tissue samples, we previously (22) found that RET tumors produce (and contain) higher concentrations of catecholamines, but secrete them at a lower rate than SDHB tumors. Our cohort was not split by the hormone synthesis metaprogram; moreover (due to a single patient) it showed a higher mean enrichment in the SDHB subset. However, it is split along the ion channel (transmembrane transport) programs that are associated with secretion (23). Metaprograms linked to neuronal development were active throughout the tumor cells, irrespective of their mutational groups.

To explore the developmental status of the tumor, we used published datasets of the developing adrenal gland as a reference. Logistic regression analysis revealed that RET-PCPG tumor cells are transcriptionally more similar to developed adrenal chromaffins, whereas SDHB-PCPG tumor cells appear to be in an earlier phase of adrenal development. Our results suggest that PCPG tumor cells had a primarily chromaffin-like phenotype, suggesting that the chromaffin cell development state may be related to mutation-associated prognosis.

In summary, we revealed extensive levels of heterogeneity among PCPG tumor cells and identified transcriptional programs related to neuronal development as key processes in these tumor cells. We speculate that in RET-PCPG, the mutation caused a development block during late chromaffin development as compared to the ‘more immature’ SDHB-PCPG tumors. To differentiate this developmental block from alternative transformative events that could also lead to modified transcriptomes of tumor cells, investigation of larger cohorts is needed. Understanding the origin of the tumor and sources of its heterogeneity may help in the development of targeted therapies.

## Data availability statement

The datasets presented in this study can be found in online repositories. The names of the repository/repositories and accession number(s) can be found below: https://ega-archive.org (EGAS00001006230, EGAS00001006230).

## Ethics statement

The studies involving human participants were reviewed and approved by CMO Regio Arnhem-Nijmegen (Gen-omgevinginteracties in de pathogenese van urologische zieken), CWOM-nr: 9803-0060. The patients/participants provided their written informed consent to participate in this study. Written informed consent was obtained from the individual(s) for the publication of any potentially identifiable images or data included in this article.

## Author contributions

PB, CR, WM, HS and HT drafted the manuscript. HT provided access to samples. PB and CR processed the samples and prepared single nuclei RNAseq libraries. PB, CR, WM and HS contributed to data analysis and interpretation. All authors contributed to the article and approved the submitted version.

## Funding

The present work was funded by the Paradifference Foundation under the coordination of Peter M T Deen (*Radboud University, Nijmegen, The Netherlands*). P.B. was supported by the Princess Maxima Center and the Paradifference Foundation. C.R.M. was supported by the Princess Maxima Center and the European Union’s Horizon 2020 Skłodowska-Curie Actions (project AiPBAND) under grant #764281. W.M. was supported by the Italian National Operational Programme on Research 2014-2020 (PON AIM 1859703-2). H.G.S. was supported by the Princess Maxima Center and KiKa (Kinderen Kankervrij).version.

## Acknowledgments

The authors thank the patients and their families for supporting this research.

## Conflict of interest

The authors declare that the research was conducted in the absence of any commercial or financial relationships that could be construed as a potential conflict of interest.

## Publisher’s note

All claims expressed in this article are solely those of the authors and do not necessarily represent those of their affiliated organizations, or those of the publisher, the editors and the reviewers. Any product that may be evaluated in this article, or claim that may be made by its manufacturer, is not guaranteed or endorsed by the publisher.

## Supplementary material

Supplementary Figure 1
**(A)** Per-sample QC metrices after filtering (UMI count: nCount_RNA, Gene count: nFeature_RNA, blue: ‘SDHB group’, red: ‘RET-group’). **(B)** UMAP visualization of the merged dataset separately annotated by patient (blue: ‘SDHB group’, red: ‘RET-group’). **(C)** Fraction of cells per sample populating the UMAP-clusters. **(D)** Fraction of cells per UMAP-clusters found per sample.

Supplementary Figure 2
**(A)** UMAP visualization of the PCPG immune cells subcluster after re-clustering (no batch-correction). The arrows point at the cells annotated as macrophages, found in tumors from similar anatomical locations. (blue: from an SDHB-tumor, red: from a RET-tumor). **(B)** UMAP visualization and relative expression levels of canonical cell type markers across the PCPG immune cell subcluster. **(C)** UMAP visualization of the PCPG stromal cell sub-cluster after re-clustering (no batch-correction). **(D)** UMAP visualization and relative expression levels of canonical cell type markers across the PCPG stromal cell sub-cluster.

Supplementary Table 1 Clinical information and snRNAseq quality parameters of processed/analyzed samples.
