# Erratum: Extensive patient-to-patient single nucleus transcriptome heterogeneity in pheochromocytomas and paragangliomas

**DOI:** 10.3389/fonc.2024.1469606

**Published:** 2024-08-01

**Authors:** Frontiers Production Office

**Affiliations:** Frontiers Media SA, Lausanne, Switzerland

**Keywords:** pheochromocytoma, neuroendcrine tumor, single cell RNA seq, transcriptome, heterogeneity, SDHB, RET, paraganglinoma

Due to a production error, the sentence “Pheochromocytoma, neuroendocrine tumor, single cell RNA-sequencing, transcriptome, heterogeneity, SDHB, RET, paraganglinoma;” was incorrectly added to the Abstract.

A correction has been made to the Abstract:

“Pheochromocytomas (PC) and paragangliomas (PG) are rare neuroendocrine tumors with varied genetic makeup and are associated with high cardiovascular morbidity and a variable risk of malignancy. The source of the transcriptional heterogeneity of the disease and the underlying biological processes that determine the outcome of PCPG remain largely unclear. We focused on PCPG tumors with germline SDHB and RET mutations, which represent distinct prognostic groups with worse or better prognoses, respectively. We applied single-nuclei RNA sequencing (snRNA-seq) to tissue samples from 11 patients and found high patient-to-patient transcriptome heterogeneity in neuroendocrine tumor cells. The tumor microenvironment also showed heterogeneous profiles, mainly contributed by macrophages of the immune cell clusters and Schwann cells of the stroma. By performing non-negative matrix factorization, we identified common transcriptional programs active in RET and SDHB, as well as distinct modules, including neuronal development, hormone synthesis and secretion, and DNA replication. Similarities between the transcriptomes of the tumor cells and those of the chromaffin- and precursor cell types suggests different developmental stages at which PC and PG tumors appear to be arrested.”

The publisher apologizes for this mistake. The original version of this article has been updated.

