# Supplementary figures and images for: Extensive patient-to-patient single nucleus transcriptome heterogeneity in pheochromocytomas and paragangliomas
Source: Front Oncol. 2022 Aug 15;12:965168. doi: 10.3389/fonc.2022.965168 (PMC9421253; doi:10.3389/fonc.2022.965168)

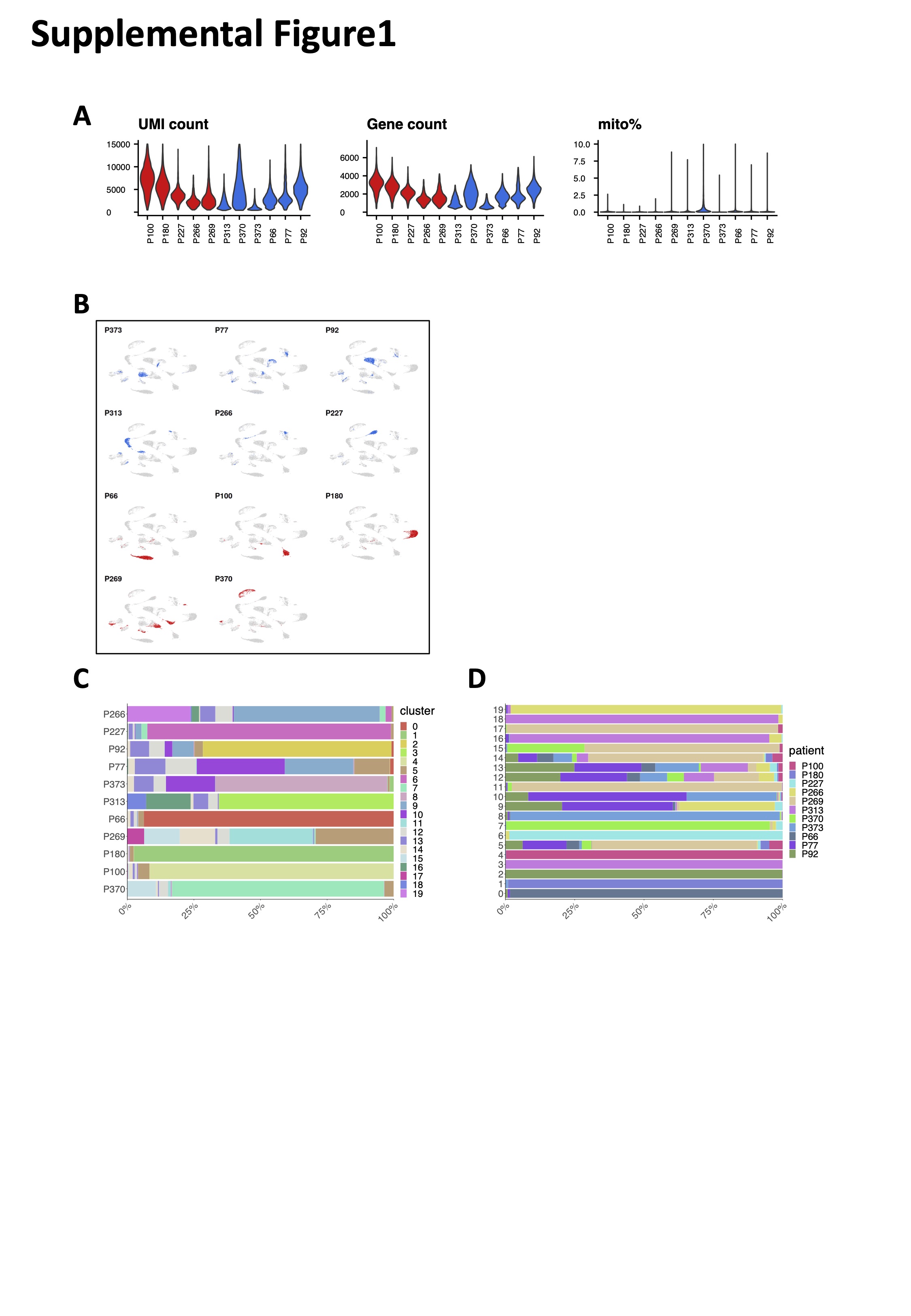

Supplement: Supplementary Figure 1 — (A) Per-sample QC metrices after filtering (UMI count: nCount_RNA, Gene count: nFeature_RNA, blue: ‘SDHB group’, red: ‘RET-group’). (B) UMAP visualization of the merged dataset separately annotated by patient (blue: ‘SDHB group’, red: ‘RET-group’). (C) Fraction of cells per sample populating the UMAP-clusters. (D) Fraction of cells per UMAP-clusters found per sample. [file Image_1.jpeg]

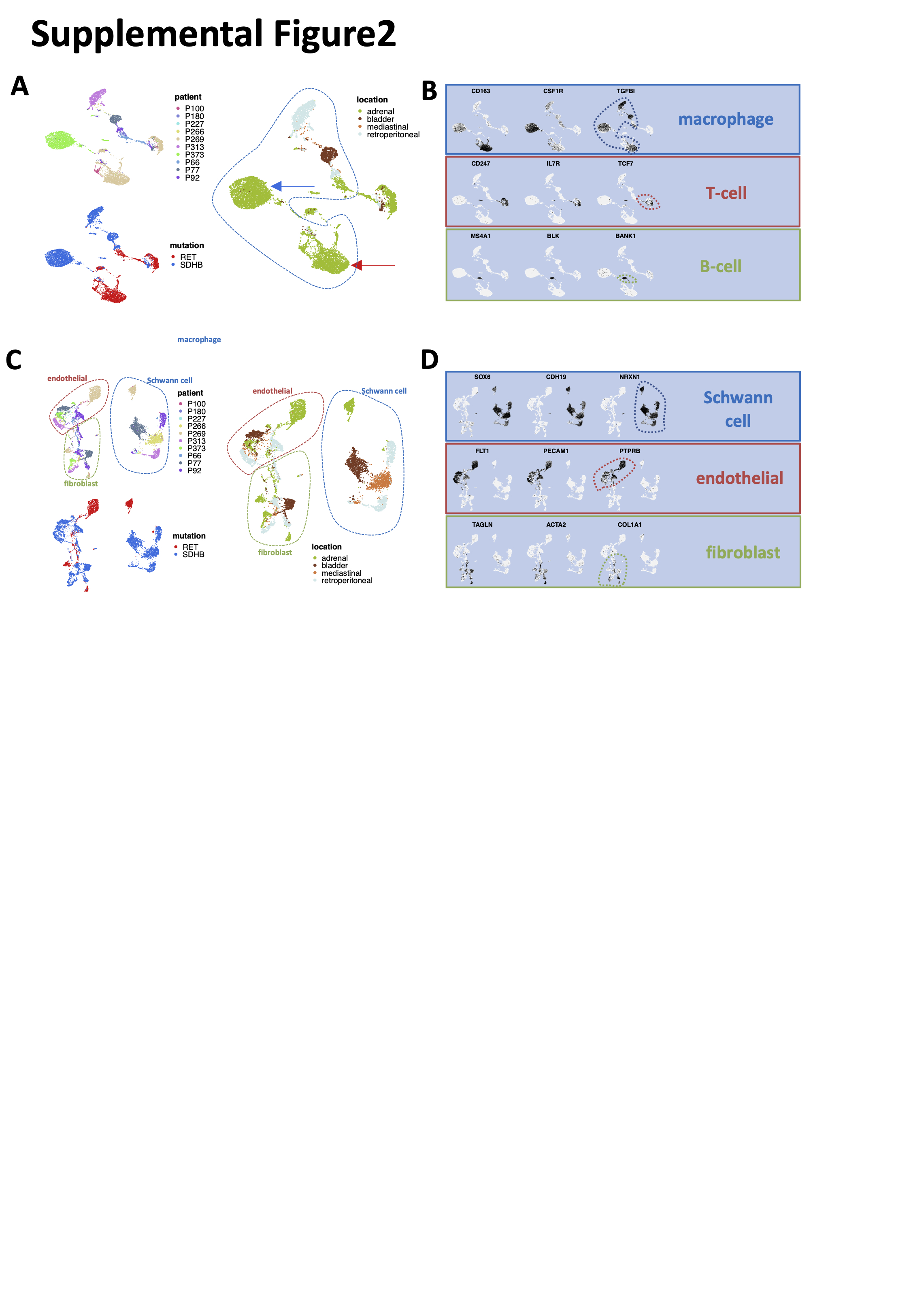

Supplement: Supplementary Figure 2 — (A) UMAP visualization of the PCPG immune cells subcluster after re-clustering (no batch-correction). The arrows point at the cells annotated as macrophages, found in tumors from similar anatomical locations. (blue: from an SDHB-tumor, red: from a RET-tumor). (B) UMAP visualization and relative expression levels of canonical cell type markers across the PCPG immune cell subcluster. (C) UMAP visualization of the PCPG stromal cell sub-cluster after re-clustering (no batch-correction). (D) UMAP visualization and relative expression levels of canonical cell type markers across the PCPG stromal cell sub-cluster. [file Image_2.jpeg]
